# Japan's global health engagement in the Western Pacific: diagnosing structural inertia and a framework for human security diplomacy

**DOI:** 10.1016/j.lanwpc.2026.101921

**Published:** 2026-07-15

**Authors:** Haruka Sakamoto, Lisa Yamasaki, Sarah Krull Abe, Satoshi Ezoe, Shinichi Egawa, Renzo R. Guinto, Hideki Hashimoto, Ataru Igarashi, Angkana Lekagul, Kaung Suu Lwin, Mitsuru Mukaigawara, Ho Namkoong, Yoshitaka Nishino, Naoki Kondo, Osamu Kunii, Santosh Kumar Rauniyar, Eiko Saito, Kenji Shibuya, Keizo Takemi, Kun Tang, Takahiro Tabuchi, Hana Tomoi, Yusuke Tsugawa, Tomoko Suzuki, Shuhei Nomura

**Affiliations:** a Graduate School of Public Health, St Luke's International University, Tokyo, Japan; b Department of Environmental Health, Harvard T.H. Chan School of Public Health, Boston, MA, USA; c Department of Emergency Medicine and Critical Care, Japan Institute for Health Security, Tokyo, Japan; d Economic Research Institute for ASEAN and East Asia (ERIA), Jakarta, Indonesia; e Department of Global Health Policy, Graduate School of Medicine, The University of Tokyo, Tokyo, Japan; f Nagasaki University, Nagasaki, Japan; g International Cooperation for Disaster Medicine Lab., International Research Institute of Disaster Science (IRIDeS), Tohoku University, Miyagi, Japan; h SingHealth Duke-NUS Global Health Institute, Duke-NUS Medical School, National University of Singapore, Singapore; i Department of Health and Social Behavior, School of Public Health, Graduate School of Medicine, The University of Tokyo, Tokyo, Japan; j Graduate School of Pharmaceutical Sciences, The University of Tokyo, Tokyo, Japan; k International Health Policy Program Foundation (IHPP Foundation), Nonthaburi, Thailand; l University of Huddersfield, Huddersfield, England, UK; m Department of Government, Harvard University, Cambridge, MA, USA; n Department of Infectious Diseases, Keio University School of Medicine, Tokyo, Japan; o Japan Center for International Exchange (JCIE), Tokyo, Japan; p Department of Social Epidemiology, Graduate School of Medicine and School of Public Health, Kyoto University, Kyoto, Japan; q Global Health Innovative Technology Fund (GHIT Fund), Tokyo, Japan; r Ocean Policy Research Institute, The Sasakawa Peace Foundation, Tokyo, Japan; s Sustainable Society Design Center, Graduate School of Frontier Sciences, The University of Tokyo, Chiba, Japan; t Medical Excellence Japan, Tokyo, Japan; u Vanke School of Public Health, Tsinghua University, Beijing, China; v Division of Epidemiology, School of Public Health, Tohoku University Graduate School of Medicine, Sendai, Japan; w The London School of Hygiene and Tropical Medicine, Department of Infectious Disease Epidemiology, Faculty of Epidemiology and Population Health, London, UK; x Nagasaki University, The School of Tropical Medicine and Global Health, Nagasaki, Japan; y Division of General Internal Medicine and Health Services Research, David Geffen School of Medicine at UCLA, Los Angeles, CA, USA; z Global Health Policy Lab, International Research Institute of Disaster Science (IRIDeS), Tohoku University, Sendai, Japan; aa Department of Health Policy and Management, School of Medicine, Keio University, Tokyo, Japan; ab Department of Food, Nutrition and Health, Graduate School of International University of Health and Welfare, Tokyo, Japan

**Keywords:** Global health governance, Human security, Japan, Western Pacific, Development assistance for health, Multipolarity, Universal Health Coverage, Health diplomacy

## Abstract

The global health architecture is under increasing strain in the context of intensifying multipolarity. Development assistance for health is declining and geopolitical fragmentation stalls collective action, leaving health sequestered within discretionary foreign aid. As Asia's sole G7 nation and a universal health coverage pioneer, Japan navigates tensions between domestic social protection and external change. Drawing on a two-round expert consultation and literature synthesis, this analysis diagnoses three interdependent structural barriers forming a cycle of strategic inertia: a crisis of solidarity, financing, and governance in the multilateral architecture; episodic leadership driven by institutional fragmentation and misaligned incentives; and unidirectional knowledge transfer that forecloses reverse learning needed to revitalise domestic capacity and regional partnerships. We propose a framework of human security diplomacy, positioning health as a pillar of regional architecture rather than discretionary expenditure. This framework advances three goals: harmonising global contribution with domestic growth; fostering solidarity and regional public goods through catalytic leadership; and mainstreaming health into social systems for planetary resilience. Central to this strategy is circular co-evolution, in which Japanese institutional expertise and partner-country innovations reinforce one another.

## Introduction

The global health architecture is fracturing in ways that slow collective decision-making and leave crucial governance functions uncoordinated.^1^^,^^2^ Global development assistance for health (DAH) peaked at US$80.3 billion in 2021 during the COVID-19 response but declined to US$49.6 billion by 2024; announced budget cuts in 2025 are projected to drive global DAH to US$38.4 billion, a level last observed in 2009.^3^ This fiscal retrenchment coincides with the United States' withdrawal from the World Health Organization (WHO), signalling a broader retreat from multilateral norm-setting. Yet the structural shift extends beyond a single country: the United Kingdom, Germany, and France have each cut Official Development Assistance (ODA) budgets under pressure from rising defence expenditure, marking a systemic reorientation of traditional donor priorities.^4^ The contraction reflects not merely post-COVID-19 fatigue but a structural divergence—a ‘momentum gap’—in which health financing has relied on voluntary, crisis-driven surges rather than sustained commitment. Unlike defence and climate policy, global health remains largely confined to discretionary foreign aid and vulnerable to recurrent cycles of crisis-driven mobilisation and retrenchment.^5^ International relations scholarship distinguishes high politics, associated with defence and sovereignty, from more discretionary domains of low politics.^6^ Although health increasingly intersects with geopolitics, trade, and economic security, it has yet to secure the durable political commitment associated with high politics.^7^

Amid this global retrenchment, Japan occupies a distinctive position in the Western Pacific, not simply as the sole G7 nation in Asia or an early pioneer of universal health coverage (UHC),^8^ but as a country navigating tensions between domestic social protection and a rapidly changing regional environment. Japan has maintained its health financing commitments even as global DAH contracted; its ODA to gross national income (GNI) ratio reached 0.39% by 2024, above the Development Assistance Committee (DAC) average of 0.33% even as the other largest donors each cut ODA in 2024,^9^ and has sought to anchor health within the evolving financial architecture through the Pandemic Fund^10^ and International Development Association (IDA) replenishment advocacy. Yet these efforts unfold within a region facing compounding pressures: an escalating non-communicable disease (NCD) burden driven by demographic ageing and inadequately regulated commercial determinants of health^11^; stalling progress toward UHC service coverage^12^; intensifying geopolitical polarisation^13^; and mounting climate-related health threats. As the Sustainable Development Goals approach their 2030 endpoint, the Western Pacific offers a critical lens on this trajectory.^3^

Despite these sustained commitments, Japan's global health engagement faces structural constraints that existing analyses have not adequately addressed.^14^^,^^15^ Prior assessments have catalogued policy outputs and financial inputs but offer limited insight into how institutional arrangements and inter-ministerial governance shape Japan's strategy,^16^ or what Japan can realistically deliver as a regional power. Yet addressing these constraints requires satisfying domestic constituencies while meeting international obligations—Putnam's two-level game in which commitments must remain ratifiable at home.^17^ This gap matters beyond Japan: a growing number of ageing high-income countries (HICs) face analogous constraints—ageing populations, rising NCD burdens, fiscal pressure on social systems,^18^ and mounting pressure to reconcile domestic with global responsibilities. Japan therefore offers a critical case for understanding how global health leadership is negotiated under demographic transition,^3^^,^^19^ constrained fiscal space, and multipolar geopolitics,^13^ all of which are increasingly shared across the Western Pacific. We approach this case through the explicit conceptual framework set out below.

This review applies a circular co-evolution framework to Japan's global health engagement, in line with the companion paper on Japan's domestic health-system reform.^20^ Drawing on reverse-innovation and reciprocal-innovation literature,^21^^,^^22^ the framework conceptualises Japan as both contributor to and beneficiary within regional networks, rather than solely as a donor and knowledge exporter.^14^^,^^23^ Japan's institutional expertise and financing strengthen partner-country capacity, while innovations developed under different constraints are absorbed to address Japan's own systemic challenges. We use this framework diagnostically to examine the institutional asymmetries producing strategic inertia and prescriptively to identify pathways through which domestic reform and international engagement reinforce one another.

This paper pursues three objectives. First, to diagnose the structural barriers that constrain Japan's capacity for sustained global health leadership, analysing how institutional incentives, budget structures, and inter-ministerial dynamics produce a reinforcing cycle of strategic inertia. Second, to assess how the circular co-evolution framework can inform mutual reinforcement between domestic reform and international engagement. Third, to propose pathways for action and consensus-building in Japan's health security diplomacy.

This paper is intended to contribute to policy dialogue among researchers, practitioners, and policymakers in Japan, the Western Pacific region, and the wider international community; the recommendations that follow are framed as priority areas for consideration rather than as directives issued on behalf of government, and their realisation will depend on the engagement of government, health system, and policy leaders whom this paper seeks to inform. This paper deliberately integrates Japan's outward-facing global health policy with inward-facing domestic policy domains such as tobacco control, insurance architecture, and long-term care. The analytical premise is that domestic credibility conditions international authority, while international commitments shape the trajectory of domestic reform. Treating these domains separately would obscure the breakdown in reciprocal learning that this paper diagnoses as a core source of strategic inertia.

Throughout, “human security diplomacy” denotes a distinct operationalisation of the United Nations (UN) conception of human security, as articulated by the 2003 report of the Commission on Human Security^24^ and codified in United Nations General Assembly Resolution 66/290 of 2012.^25^ It deploys health as a global public good to co-construct regional governance, moving beyond human security as an ODA framework toward its application as an instrument of regional architecture.

## Methods

We assembled a multidisciplinary working group of 25 specialists across health policy, diplomacy, and international development. Two sequential survey rounds were analysed using inductive thematic analysis following Braun and Clarke.^26^ Round 1 mapped structural challenges in Japan's global health strategy and Round 2 identified forward-looking goals and policy actions. Outputs were consolidated into the resulting tables and contextualised through an integrative literature review. Full survey instruments, coding procedures, and analytical audit trails are provided in the Supplementary Methods.

### Search strategy and selection criteria

The literature search was designed as an integrative review to contextualise and substantiate the findings generated by the working group. A structured search was performed in PubMed for all publications from database inception to January 15, 2026, supplemented by hand-searching of reference lists of key reviews and by targeted searches of official policy documents and grey literature from government agencies, intergovernmental bodies, and health diplomacy forums in the Western Pacific. Free-text and MeSH terms included combinations of ‘global health’, ‘global health governance’, ‘global health strategy’, ‘Universal Health Coverage’, ‘health diplomacy’, ‘health security’, and ‘political economy of health’. Publications and policy documents in English and Japanese were reviewed to ensure comprehensive coverage. Titles and abstracts identified through the searches were screened by two authors, with disagreements resolved through discussion where necessary. We included publications and other sources that were judged to be relevant and informative for the aims of this Review. This paper is an integrative narrative review grounded in the structured expert consultation.

### Role of the funding source

The funder of the study had no role in study design, data collection, data analysis, data interpretation, or writing of the report.

## Diagnosis: three interdependent structural barriers

The three structural barriers described in this section were derived from the coding and aggregation of Round 1 consultation responses and were substantiated against the integrative literature review; the coding and consensus procedure linking Round 1 responses to these barriers is described in the Supplementary Methods (Section 2.4). Our analysis identified three interdependent structural challenges (Table 1). These operate at the global level (a crisis of solidarity, financing, and governance in the multilateral architecture), the regional level (the limits of unidirectional knowledge transfer), and the national level (the domestic bureaucracy of episodic leadership). The three are not merely concurrent; they form a reinforcing cycle of strategic inertia. Global instability heightens the need for coordinated responses that fragmented domestic institutions cannot sustain, while unidirectional engagement forecloses the reverse learning that could revitalise both domestic capacity and regional partnerships. As each challenge amplifies the others, the resulting governance vacuum leaves many states in the Western Pacific to navigate an increasingly contested landscape of competing regional interests with diminishing predictability in institutional support.

**Table 1:** Key structural challenges of Japan's global health strategy.

Structural Challenge Category	Specific Critical Challenges
A. The shifting global health architecture and the crisis of solidarity	**Solidarity crisis:** Geopolitical fractures and ‘border-first’ policies erode the trust essential for multilateral cooperation. **Financing retrenchment:** A precipitous decline in DAH threatens the sustainability of health systems in LMICs. **Governance paralysis:** Geopolitical fragmentation has stalled collective action, leaving global institutions unable to manage acute crises. **Unregulated threats:** Governance gaps leave the world exposed to multifaceted threats, including the commercial determinants of health and climate change.
B. Japan's fragmented policy and the structural roots of leadership challenges	**Episodic leadership:** Diplomacy relies on high-visibility events (e.g., G7) rather than sustained engagement, reflecting a long-standing decoupling of political momentum from contributions and limiting long-term impact. **Coordination deficit:** The Headquarters for Healthcare Policy coordinates across ministries (e.g. MOFA, MHLW) but lacks the binding authority to enforce a cohesive whole-of-government strategy. **Moving targets:** Frequent leadership changes disrupt policy continuity. **Role confusion:** Japan clings to a traditional donor model, failing to pivot to a necessary role as a proactive rule-shaper in multipolar Asia. **Rhetoric-reality gap:** Domestic policy implementation lags behind international rhetoric on agendas like climate and sustainable systems, undermining the normative legitimacy of Japan's global health leadership. **Political indifference:** Global health lacks domestic political currency, hindering the will to overcome institutional fragmentation.
C. Breakdown in the ‘circular model’: missed opportunities for mutual growth	**Unidirectional flow:** The strategy prioritises exporting domestic models (‘teaching’) over co-creation and mutual learning from the Global South. **Domestic-global disconnect:** Domestic policies and systems are designed in isolation from global dynamics, missing opportunities to ‘reverse import’ agile innovations from regional partners. **AHWIN gap:** The Asia Health and Wellbeing Initiative has yet to translate its bidirectional ambition into systematic inbound learning.

Three interdependent structural barriers were identified through the survey analysis; each reinforces the others, collectively creating a cycle of strategic inertia.

DAH = Development Assistance for Health; LMICs = low- and middle-income countries; G7 = group of 7; MOFA = Ministry of Foreign Affairs; MHLW = Ministry of Health, Labour and Welfare; AHWIN = Asia Health and Wellbeing Initiative.

Items in this table were derived from the coding and aggregation of Round 1 working group responses, following the procedure described in the Methods, and were substantiated against the integrative literature review. The coding and consensus procedure that produced these three structural challenge categories from Round 1 responses is described in Supplementary Methods (Section 2.4).

China's expanding role in health governance, including the Health Silk Road and vaccine diplomacy, further complicates the regional landscape and requires engagement that balances regional cooperation with rules-based governance.^27^, ^28^, ^29^

### The shifting architecture: a crisis of legitimacy and trust

The fracturing of the global health architecture reflects deeper strains in multilateral cooperation and global solidarity.^30^ Working group members consistently identified this erosion of multilateral trust as the defining feature of the current moment, and the literature synthesis corroborates the pattern across recent analyses of global health diplomacy under multipolarity. The multilateral architecture forged after World War II, the constellation of institutions, norms, and financing mechanisms that underpinned decades of health cooperation, is buckling under intensifying multipolarity. Unlike the Cold War's bipolar stability, the current fragmentation is diffuse and unpredictable, eroding the mutual trust on which health cooperation depends.^13^ Recent border-first policy shifts have weakened confidence in international institutions' ability to mediate competing interests and provide predictable, long-term support,^31^ widening a structural momentum gap that leaves health particularly exposed. Unlike defence or climate, health has yet to generate the domestic constituencies or protected budget lines necessary to withstand fiscal retrenchment.

The financing consequences are severe. Global health expenditure remains trapped in crisis-driven surges that hollow out institutional capacity between emergencies, and the current retrenchment is uniquely severe. Projected declines in DAH far exceed historical norms, with modelling studies suggesting profound consequences for infectious disease control, immunisation, and maternal and child health in low-income countries.^3^ This funding volatility is compounded by governance paralysis: geopolitical fragmentation has stalled collective action, leaving critical governance functions, from pandemic treaty negotiations to antimicrobial resistance (AMR) frameworks, without the political consensus required for progress.

In the Western Pacific, these global pressures are compounded by an escalating burden of NCDs.^11^ Unchecked commercial determinants of health, including the aggressive marketing of ultra-processed foods and tobacco, combined with rapid population ageing, have driven NCDs to account for the majority of the regional disease burden.^32^ Although overall progress towards UHC has continued, NCD-related service coverage has lagged markedly, with the NCD sub-index now representing the weakest component of service coverage in the region.^11^

**Fig. 1:**
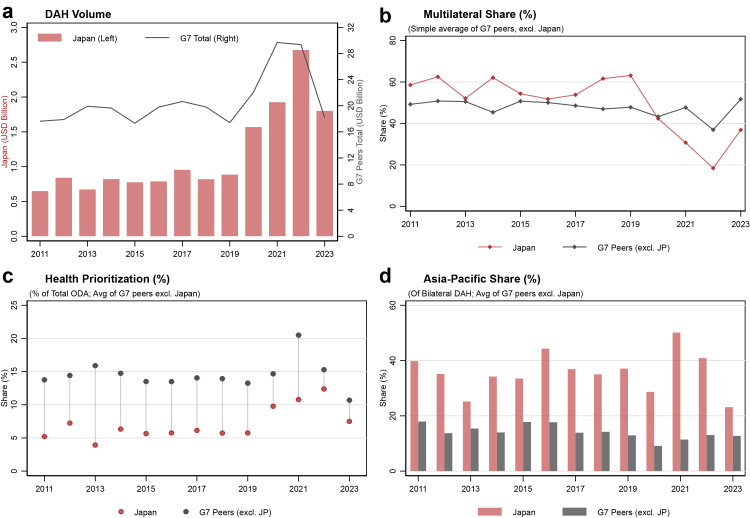
**Structural divergence in Japan's global health financing, 2011–2023.** Panel (a) shows global DAH volume in constant 2023 US$ billion: Japan (red bars, left axis) is compared against the combined total of other G7 members (grey line, right axis), highlighting both the scale difference and the pandemic-era surge. Panels (b), (c), and (d) show proportional indicators: the share of DAH channelled through multilateral institutions, health prioritisation defined as the percentage of DAH within total official development assistance, and the share of bilateral DAH allocated to the Asia–Pacific region, respectively. For panels (b), (c), and (d), Japan is compared with the simple unweighted average of G7 peers excluding Japan. Only panel (d) concerns the regional distribution of DAH flows; the other panels concern global DAH volume or proportional characteristics of DAH portfolios. Data source: Visualizing ODA (VODA). 2025. https://visualizingoda.org/ (accessed February 10, 2026).

Against this volatile backdrop, Japan's financing follows a distinctive pattern that diverges from most G7 peers, a divergence that carries both regional strengths and structural limitations. As Fig. 1 shows, Japan's DAH profile remains heavily bilateral and regionally concentrated. Panel (a) compares DAH volume against G7 peers; panels (b)–(d) show proportional indicators (multilateral share, health prioritisation, Asia–Pacific concentration). Although Japan increased DAH during COVID-19, much of the expansion relied on bilateral yen loans rather than multilateral pooled mechanisms. Consequently, Japan's multilateral share of DAH fell below the G7 average, while the share of health within total ODA remained comparatively low. This configuration sustains regional influence but limits Japan's leverage within collective governance platforms.

The regional concentration, however, also represents an unrealised strategic asset that provides the foundation for the sustained partnership with Western Pacific neighbours that the co-evolution framework seeks to build upon.

### Japan's fragmented policy: the structural roots of leadership challenges

Japan's global health leadership appears episodic because its consistent day-to-day contributions are often decoupled from the high-level political momentum of summits such as the G7, G20, and the Tokyo International Conference on African Development (TICAD) (Table 1). The working group characterised this pattern as event-driven engagement shaped by institutional incentives. The 2019 G20 Osaka Summit's first-ever joint Finance and Health Ministers' Session,^33^ which catalysed the UHC Knowledge Hub established in 2025, illustrates how summit diplomacy can generate durable governance innovations when backed by sustained follow-through. Yet the structural concern is not event-driven leadership per se but the absence of mechanisms to lock in and operationalise the commitments generated at such moments. The absence of domestic constituencies for global health is compounded by rising fiscal pressures, under which multilateral health investments are often perceived as delivering diffuse or indirect national benefits compared with the visible returns associated with bilateral engagement.^34^^,^^35^ Without a clear narrative linking global public goods to direct national interests, political incentives favour high-visibility announcements over the long-term system strengthening required for sustained leadership (Panel 1).

Panel 1 **Case study: contrasting models of engagement in the Western Pacific**

How do different modes of engagement shape the sustainability of health system support in the Western Pacific?

• Debates on ‘episodic’ versus ‘strategic’ engagement often attribute differences to donor intent, yet two distinct institutional logics better explain the contrast. Event-driven engagement denotes summit-anchored, time-bound mobilisation that generates political visibility but whose follow-through depends on subsequent political attention. Such moments reflect what has been described as the reconfiguration of power and negotiation arenas in contemporary global health diplomacy, where high-level political forums, such as the G7 and G20, serve as sites for agenda-setting yet remain vulnerable to shifting geopolitical priorities.^13^ By contrast, long-horizon, institutionally embedded cooperation—sustained through multi-year commitments, embedded personnel, and shared operational protocols—aligns with health system strengthening and resilience frameworks that emphasise institutional capacity and continuity.^36^^,^^37^ Neither is a fixed national attribute; both coexist within a single donor's portfolio.
• Japan's engagement has frequently been anchored to the former logic: the G7 Ise-Shima Summit (2016) consolidated universal health coverage (UHC) as a global norm,^38^ the G20 Osaka Summit (2019) convened the first joint Finance and Health Ministers' Session, and the G7 Hiroshima Summit (2023) advanced pandemic preparedness architecture.^10^ These summits generated important agenda-setting effects.^13^^,^^39^ However, when commitments are embedded within short political cycles rather than institutionalised in multi-year frameworks, sustaining implementation momentum can prove challenging. Evidence from analyses of donor transitions and health financing reforms underscores the importance of predictable, multi-year commitments and early institutional integration for maintaining coverage and enabling effective national planning.^40^^,^^41^
• Programmes built on the latter logic demonstrate different outcomes. The Mekong Basin Disease Surveillance network (MBDS)—linking six countries through embedded technical personnel, shared protocols, and multi-year commitments—sustains regional health security independently of any single donor's political cycle.^42^ Japan's own Japan International Cooperation Agency (JICA) programmes in primary care strengthening similarly show how engagement maintained over decades builds the operational continuity on which partner nations depend.^43^
• The structural concern is therefore not event-driven leadership per se—agenda-setting remains indispensable—but the absence of mechanisms that translate episodic commitment into durable institutional arrangements.

Several institutional features reinforce this pattern, as summarised in Table 1: most notably a coordination forum that produces administrative co-presence rather than binding authority. The Headquarters for Healthcare Policy, the inter-ministerial body that adopted the 2022 Global Health Strategy and oversees Japan's global health agenda, illustrates this: it operates by consensus without independent fiscal or directive authority.^14^ Finally, inconsistencies between international commitments and domestic implementation, notably Japan's tobacco control record,^44^ erode the normative credibility on which Japan's global health leadership depends. Japan has prominently positioned NCD prevention within G7 and G20 diplomacy,^10^ yet domestic implementation of WHO Framework Convention on Tobacco Control (FCTC) measures remains limited, while the government retains a one-third stake in Japan Tobacco Inc.^44^, ^45^, ^46^, ^47^ This gap between international advocacy and domestic regulation weakens Japan's credibility in promoting regional regulatory harmonisation (Panel 2). Together, these constraints lock Japan into an event-driven equilibrium in which leadership peaks during diplomatic moments but recedes thereafter.

Panel 2 **The political economy of Japan's ‘episodic’ leadership**

Why does Japan's global health leadership repeatedly surge around high-visibility events, yet struggle to sustain momentum?

• Japan's ‘episodic’ engagement is best understood as a product of its domestic political economy rather than a lack of technical capacity. Analyses of political priority in global health emphasise that sustained attention depends on actor cohesion, institutional authority, and governance incentives that extend beyond crisis windows.^39^ Three structural dynamics, each individually rational, combine to sustain the pattern.
• Japan's global health policymaking is distributed across several institutions. The Ministry of Foreign Affairs (MOFA) is responsible for diplomacy and the administration of official development assistance. The Ministry of Health, Labour and Welfare (MHLW) is responsible for domestic system stewardship and for the Global Health Vision that articulates the ministry's international priorities. The Ministry of Economy, Trade and Industry (METI) is responsible for industrial competitiveness, including the international dimensions of the pharmaceutical and medical technology sectors. The Ministry of Finance (MOF) is responsible for fiscal discipline across budget envelopes. Bilateral programme implementation is primarily undertaken through the Japan International Cooperation Agency (JICA). The Headquarters for Healthcare Policy, housed in the Cabinet Secretariat under the direct authority of the Prime Minister, is the inter-ministerial coordination body that adopted the 2022 Global Health Strategy and, in principle, oversees coherence across this policy architecture.
• First, ministerial mandates generate competing optimisation logics. Although MOFA, MHLW, METI, MOF, JICA, and the Headquarters for Healthcare Policy are formally connected through the 2022 Global Health Strategy, the strategy does not establish binding budgetary integration or joint accountability mechanisms.^48^ Each institution is evaluated against its own mandate, so cross-institutional alignment—which requires shared authority, pooled resources, and sustained personnel coordination—carries institutional costs that no single actor has sufficient incentive to absorb.
• Second, coordination forums tend to produce administrative co-presence rather than strategic cohesion. The Headquarters for Healthcare Policy serves as an inter-ministerial platform, yet operates through consensus without independent fiscal authority or directive power.^48^ Comparative analyses of global health governance note that institutional foreground commitments often exceed the coordinating capacity embedded in background practices.^49^ In this configuration, responsibility is distributed without concentrating decision-making power, limiting consolidation of multi-year strategy.
• Third, global health lacks a durable domestic political constituency. Unlike defence or industrial policy, which are anchored to electoral incentives or sectoral interests, global health generates diffuse, long-horizon benefits that are difficult to attribute politically. Studies of political priority formation show that issues without concentrated constituencies struggle to sustain salience once crisis attention recedes.^39^ In fiscally constrained environments, such issues are especially vulnerable to retrenchment.^40^
• Together, these dynamics lock Japan into an event-driven equilibrium: summit mobilisation proceeds because it aligns with existing institutional incentives and requires no structural redistribution of authority. The constraint is therefore not capacity but institutional architecture.

### Limitations of the circular model: the challenge of unidirectional knowledge flow

The third structural challenge concerns the limitations of unidirectional knowledge transfer. Working group members repeatedly returned to this asymmetry as the least-recognised of the three structural barriers, and the integrative literature review confirmed a paucity of institutional mechanisms for systematic reverse learning. Japan has historically exported its health system expertise to partner countries across the region.^14^^,^^50^ Yet the predominant flow of knowledge has remained one-directional, with few institutional pathways for Japan to absorb innovations from other contexts. The Asia Health and Wellbeing Initiative (AHWIN), launched to promote such bidirectional exchange, has yet to translate this ambition into systematic inbound learning.^51^

**Fig. 2:**
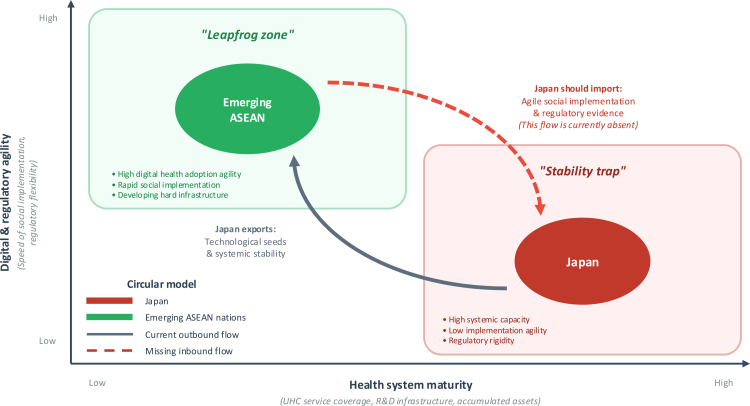
**The innovation complementarity map for circular co-evolution.** This figure operationalises the circular co-evolution framework defined in the Introduction. The horizontal axis represents health system maturity (encompassing progress toward universal health coverage (UHC), research and development (R&D) infrastructure, and accumulated assets). The vertical axis represents digital and regulatory agility (encompassing speed of social implementation and regulatory flexibility). Japan is positioned in the ‘stability trap’ (bottom-right), characterised by high systemic capacity, low implementation agility, and regulatory rigidity. Emerging Association of Southeast Asian Nations (ASEAN) economies occupy the ‘leapfrog zone’ (top-left), characterised by high digital health adoption agility, rapid social implementation, and developing hard infrastructure. The solid arrow represents the current outbound flow, namely Japan's export of technological seeds and systemic stability to the region. The dashed arrow represents the missing inbound flow, namely the agile social implementation and regulatory evidence that Japan should import from the region. The three recommendation domains of Table 2 operationalise this framework as follows. Recommendation 1, human security diplomacy, constructs the political authority under which bidirectional flows can be legitimised. Recommendation 2, institutional and digital integration, builds the infrastructural channels through which innovations move in both directions. Recommendation 3, impact-based financing, aligns incentives so that reverse learning is rewarded rather than penalised. This conceptual mapping highlights structural complementarities rather than precise country rankings.

This asymmetry reflects a gap between technological capacity and implementation agility. Regional partners have developed innovative digital-health models relevant to Japan's own systemic challenges,^52^ yet domestic regulatory barriers impede adoption. Fig. 2 visualises this structural complementarity: Japan occupies a stability trap of high systemic capacity but lower implementation agility,^53^ while several emerging Association of Southeast Asian Nations (ASEAN) nations occupy a leapfrog zone of higher digital and regulatory agility. Without reciprocal channels, mutual learning that could strengthen both Japan's domestic resilience and regional partnerships remains unrealised.

## Vision 2040: a strategic pivot to human security diplomacy

The three goals presented in this section synthesise the forward-looking priorities identified by working-group members in Round 2 of the consultation, refined through iterative discussion and grounded in the evidence assembled through the integrative literature review. The derivation of each goal from the Round 2 coded concepts, and the mapping of Round 2 action items to the goal-action matrix, are provided in Supplementary Table S1. We propose that Japan redefine health as a pillar of human security diplomacy, extending its long-standing tradition of protecting and empowering individuals.^24^ Japan's 2022 Global Health Strategy identifies health security and UHC as its two principal pillars; the three goals below reframe these commitments within a broader normative architecture. In this vision, health serves simultaneously as a prerequisite for economic security,^54^ a vehicle for regional solidarity, and a foundation for planetary resilience.^55^

In an interconnected world, Japan's domestic stability is inseparable from the resilience of its regional partners; the question is not whether to invest in global health, but how to structure that investment so that domestic reform and international engagement reinforce one another.^56^ This reframing carries a deliberate tension. An economic-security lens risks instrumentalising health—for instance, by privileging domestic industries over equitable regional access.^57^ Yet the alternative, treating global health as discretionary expenditure divorced from national interest, has produced precisely the episodic engagement documented above. As the post-war liberal international order buckles, the framework advanced here positions sustained cooperation as a rational imperative, anchored by three interdependent goals whose logic is cumulative (Table 2). Each addresses one of the structural barriers diagnosed above. The three goals correspond to the structural barriers identified above: domestic fragmentation, multilateral instability, and unidirectional knowledge flow. Together, they aim to elevate health to the level of high politics through sustained institutional and regional leadership.

**Table 2:** Goal-action matrix for Japan's strategic pivot.

Goals/Actions	1. Human security diplomacy to maximise authority and shape health governance	2. Operationalise co-evolution through institutional and digital integration	3. Transition to impact-based financing for accountability and equity
Goal 1: Harmonise global contribution with domestic growth through human security diplomacy	• Upgrade the Headquarters for Healthcare Policy with budget coordination authority and personnel exchange capacity to enforce consistent diplomacy across MOFA, MHLW, and other relevant agencies • Fuse economic security logic into health strategies to position global health as a core pillar of national and global stability	• Pivot from unidirectional technology export to reciprocal innovation by reinvigorating AHWIN toward operational co-evolution, integrating mHealth and AI-diagnostics from Southeast Asia • Leverage comprehensive legislative systems (e.g., NCD control, universal insurance) to build mutually compatible and safe regulatory environments across the region • Build transnational innovation ecosystems linking researchers, regulators, and entrepreneurs to co-develop solutions for regional and domestic health priorities	• Rewrite evaluation frameworks to shift from inputs to outcomes, using metrics such as DALYs averted, UHC service coverage expansion, and regulatory harmonisation • Focus on long-term trajectories of health system strengthening and broader impacts on climate and environment to overcome attribution challenges • Incorporate strategic access provisions in public R&D funding, encouraging voluntary technology transfer or tiered pricing for LMICs
Goal 2: Foster solidarity and regional public goods through catalytic leadership	• Lead regional solidarity in regulating CDoH through catalytic leadership • Harmonise regulatory frameworks to address CDoH and lead creation of regional benchmarks to prevent regulatory arbitrage • Lead development of regional quality standards and shared manufacturing protocols to secure pharmaceutical supply chains • Extend ASEAN PPWG mandate to antimicrobial stewardship and supply-chain auditing, and develop PMAS into a proactive pharmacovigilance network for AMR surveillance and pandemic early-warning • Build on the G7 Hiroshima AI Process to convene regional standards for AI-enabled health technologies and lead coordinated action against health-related misinformation and disinformation	• Create a regional digital interoperability framework to enable secure exchange of health data for shared intelligence and ensure the portability of health records as workers and patients move across borders • Advance mutual recognition of professional qualifications, building on ASEAN's MRA, to foster circular workforce mobility as a regional public good	• Integrate fiscal scale of MDBs (World Bank, ADB) with technical mandates of WHO and regional health frameworks (e.g., ASEAN, PIF) to align fragmented investments • Support domestic resource mobilisation by strengthening Public Financial Management (PFM) and administrative integrity in partner nations • Operationalise the UHC Knowledge Hub as a platform for financial governance models, integrating planetary health perspectives
Goal 3: Mainstream health into social systems for planetary resilience	• Leverage expertise in industrial pollution control and disaster-resilient infrastructure to champion integration of DRR into primary healthcare infrastructure • Operationalise health metrics across transport, energy, agriculture, and urban planning sectors through a Health in All Policies approach • Embed health metrics in existing DRR partnerships, including the ARCH Project, as a pragmatic entry point for cross-sectoral HiAP expansion	• Co-develop and deploy adaptive technologies for climate-resilient health infrastructure as a shared regional asset • Establish joint R&D platforms for climate-adaptive health technologies and negotiate regional pre-positioning agreements for essential medical supplies in disaster-prone areas • Develop common technical specifications for climate-health early-warning systems enabling interoperable alert and response across national borders	• Secure international resources for climate-health intelligence systems providing early warning of environmental anomalies • Target system-building support for nations with limited crisis response capacity to prevent regional escalation • Anchor a regional public good learning platform via the UHC Knowledge Hub to systematically share lessons from successes and failures

Rows list the three strategic goals; columns list the three action domains through which each goal is operationalised. Each cell identifies the specific actions that advance the corresponding goal within that action domain.

MOFA = Ministry of Foreign Affairs; MHLW = Ministry of Health, Labour and Welfare; AHWIN = Asia Health and Wellbeing Initiative; CDoH = commercial determinants of health; NCD = non-communicable disease; MDBs = multilateral development banks; ADB = Asian Development Bank; WHO = World Health Organization; ASEAN = Association of Southeast Asian Nations; PIF = Pacific Islands Forum; PFM = public financial management; DRR = disaster risk reduction; DALYs = disability-adjusted life years; R&D = research and development; UHC = universal health coverage; LMICs = low- and middle-income countries; AI = artificial intelligence; AMR = antimicrobial resistance; ARCH = ASEAN Regional Capacity on Health Development; HiAP = Health in All Policies; MRA = Mutual Recognition Arrangements; PPWG = Pharmaceutical Product Working Group; PMAS = Post-Market Alert System; mHealth = mobile health.

Each of the three goals primarily addresses one of the three structural barriers diagnosed in Table 1. Goal 1, harmonising global contribution with domestic growth through human security diplomacy, addresses Barrier B (Japan's fragmented policy and the structural roots of leadership challenges). Goal 2, fostering solidarity and regional public goods through catalytic leadership, addresses Barrier A (the shifting global health architecture and the crisis of solidarity). Goal 3, mainstreaming health into social systems for planetary resilience, addresses Barrier C (breakdown in the circular model), while also engaging cross-sectoral dimensions that extend beyond the health sector. The three action domains (columns) are not specific to any single goal; rather, every goal is advanced through a combination of human security diplomacy, institutional and digital integration, and impact-based financing. Each cell therefore specifies the subset of actions through which the row goal is operationalised through the column action domain. Each cell integrates action items generated in Round 2 of the working group consultation with evidence from the integrative literature review, so that the goal-action matrix serves as both a synthesis of expert judgement and a summary of the literature-derived evidence base. The full mapping from coded Round 2 concepts to individual cells of this matrix is provided as Supplementary Table S1.

### Goal 1: harmonise global contribution with domestic growth through human security diplomacy

The human security diplomacy framework advanced above dissolves the separation of national interest from international contribution—a structural source of episodic engagement diagnosed above—by treating health as integral to economic security. Regional health security constitutes a public good whose under-provision threatens Japan's domestic resilience through supply-chain disruptions and cross-border threats. By demonstrating that regional engagement directly strengthens domestic resilience, this reframing addresses the core tension in Putnam's two-level game. The paradigm this entails is not linear technology transfer but circular co-evolution.^21^

This cycle is not hypothetical. Panel 3 documents how Thailand, the Philippines, Indonesia, and Viet Nam have each developed health system innovations—in payment design, digital service delivery, and community-based care—that directly address challenges Japan now faces. These cases demonstrate that substantive precedents for reverse learning already exist across the Western Pacific, yet converting this latent complementarity into a sustained reciprocal cycle requires the institutional mechanisms proposed in the recommendations below.

Panel 3 **Concrete precedents for circular co-evolution in the Western Pacific**

The structural complementarity visualised in Fig. 2—Japan's high systemic capacity paired with the implementation agility of emerging regional partners—is not merely theoretical. Operational programmes across the Western Pacific demonstrate bidirectional learning at multiple levels of health system interaction.

• At the policy and governance level, Thailand's Universal Coverage Scheme, developed with technical cooperation from Japan and other partners, adopted strategic purchasing mechanisms that strengthened cost discipline and incentivised primary care performance.^58^ The National Health Security Office centralised purchasing authority and applied capitation and diagnostic-related group payments within a global budget, generating efficiency gains and equity improvements that extended beyond their original design.^59^ The institutional adaptations Thailand developed in implementing these mechanisms now represent innovations relevant to cost containment and purchasing reform debates in high-income systems, illustrating how recipient countries can evolve original models in ways that offer reverse learning opportunities for the original knowledge contributions.
• At the service delivery level, Indonesia and the Philippines have expanded primary care reach through integration of digital platforms and mixed public–private delivery models within national insurance frameworks.^60^ Rapid regulatory adaptation during health emergencies demonstrated the capacity of these systems to incorporate teleconsultation, pharmacy distribution networks, and remote triage into publicly financed care pathways. Such regulatory agility offers institutional design lessons for Japan, where digital consultation remains administratively separated from the mainstream social insurance infrastructure.
• At the workforce and system design level, Viet Nam's strengthening of primary care—supported over decades by external technical cooperation—has integrated community-level providers into non-communicable disease screening and chronic disease management.^43^ Long-term investment in district-level capacity and workforce distribution created adaptive space for extending NCD services beyond hospitals.
• Taken together, these cases demonstrate a recurring pattern: Japanese cooperation contributed to foundational capacity, while partner countries subsequently adapted and extended institutional designs in ways that now offer reverse-learning opportunities. Without structured channels to capture such feedback loops, complementarity remains episodic rather than systematised.

### Goal 2: foster solidarity and regional public goods through catalytic leadership

This goal operationalises the provision of health as a global public good through the deliberate construction of regional public goods. These include harmonised regulatory standards, pooled surveillance networks, interoperable digital health infrastructure, and shared pharmaceutical quality protocols; the regional scale is where diffuse global norms become tractable, and where Japan's geographic, institutional, and diplomatic assets can generate disproportionate leverage. Circular co-evolution at the bilateral level cannot succeed without a stable regional environment. As geopolitical tensions strain multilateral institutions and traditional donors retrench, Japan's role must evolve from traditional donor to regional strategic architect. The contraction of ODA from major Western partners^61^ has intensified pressure to strengthen domestic resource mobilisation for health, a trajectory increasingly visible in countries such as Senegal.^62^ This requires institutional design rather than hierarchical authority or financial dominance alone, grounded as much in solidarity as in self-interest.^63^^,^^64^

Existing regional institutions, though fragmented, provide nascent foundations for this catalytic role, including WHO Regional Offices, ASEAN, and the Free and Open Indo-Pacific (FOIP) framework^65^ through which health can be embedded as a pillar of regional security. Yet these institutional seeds have not yet coalesced into the coherent governance architecture that a multipolar Western Pacific demands. The recommendations below identify platforms through which Japan can operationalise catalytic leadership, transforming isolated institutional assets into a coordinated regional framework in which diverse national systems co-innovate governance architectures that integrate health with other policy domains—making solidarity the most effective form of collective security.

Two forces further complicate this agenda: the accelerating deployment of artificial intelligence (AI) in health, which presents both opportunity and systemic risks of technological dependency and algorithmic bias; and the proliferation of health-related misinformation that undermines the trust on which regional cooperation depends. Japan can help shape regional norms for AI governance and information integrity.

### Goal 3: mainstream health into social systems for planetary resilience

The co-evolution and regional solidarity advanced in Goals 1 and 2 must ultimately confront a reality that extends beyond the health sector. In the Anthropocene, human health is inseparable from the integrity of planetary systems.^66^ The 2025 Planetary Health Check confirms that seven of nine planetary boundaries have now been transgressed, with ocean acidification breached for the first time.^67^ At the national level, downscaled analyses further indicate that high-income countries, including Japan, transgress nearly all of the planetary boundaries allocated to them while meeting most social-foundation thresholds, whereas a number of regional partners, including Viet Nam, approach more closely the “safe and just space” between social shortfall and ecological overshoot.^68^ This asymmetry positions Japan not only as a contributor of accumulated expertise but as a learner from constrained development pathways that have remained closer to planetary limits. These transgressions pose cascading risks to human health through disrupted food systems, degraded water quality, expanded vector-borne disease ranges, and compound disasters.^55^ These threats cannot be addressed by the health sector alone; they demand cross-sectoral governance architectures that embed health considerations into energy, agricultural, and urban planning decisions. How Japan's domestic expertise is translated into regional norm-setting and cross-sectoral governance is a question of implementation addressed in the recommendations that follow.^66^

## From diagnosis to action: pathways for consensus-building and strategic action

The three goals outlined above define the normative architecture of Japan's strategic pivot; translating this architecture into practice requires a corresponding set of actionable recommendations. Table 2 organises these recommendations as a goal-action matrix, mapping each goal against three action domains, namely human security diplomacy (Recommendation 1), institutional and digital integration (Recommendation 2), and impact-based financing (Recommendation 3), so that every cell identifies a specific, high-priority intervention. Although these recommendations are directed primarily at Japan's policy community, the circular co-evolution model on which they rest is inherently reciprocal: their realisation depends on the active engagement of regional partners, whose complementary roles are noted where relevant in the recommendations that follow.

### Recommendation 1: human security diplomacy to maximise authority and shape health governance

The diagnosis section identified ministerial fragmentation, the decoupling of political momentum from technical engagement, and the credibility gap between international advocacy and domestic policy as core drivers of Japan's cycle of strategic inertia. Recommendation 1 addresses these barriers by constructing an enduring, whole-of-government diplomacy that systematically projects Japan's domestic institutional strengths into regional and global norm-setting. Concretely, elevating health to the status of high politics, comparable to defence and economic security, requires three changes that Recommendation 1 operationalises. First, protected multi-year budget envelopes insulated from discretionary cuts, modelled on defence and disaster-resilience financing, so that global health commitments are not the first casualty of fiscal retrenchment. Second, prime-ministerial-level accountability anchored in a Cabinet Secretariat mechanism with binding coordination authority, so that political commitment outlasts individual summits and ministerial turnover. Third, the explicit linkage of global and regional public goods to identifiable national interests, including supply chain security, demographic sustainability, pandemic preparedness, and strategic autonomy, so that investment in global public goods is politically defensible at home. In this framework, regional public goods, as discussed in the sections ‘Lead regional solidarity against comprehensive health security threats’ and ‘Mainstream planetary health through cross-sectoral diplomacy,’ are the primary vehicle through which the abstract category of a global public good is made tractable and politically legible; by building enforceable regional arrangements, Japan simultaneously contributes to the global commons and anchors health within the domestic political logic of high politics.

#### Unify diplomacy via whole-of-government coordination

The Headquarters for Healthcare Policy coordinates through consensus but lacks the binding authority to set cross-ministerial priorities or allocate resources. Upgrading the Headquarters by granting genuine budget coordination authority and the capacity to direct personnel exchanges across the Ministry of Foreign Affairs (MOFA), the Ministry of Health, Labour and Welfare (MHLW), the Ministry of Economy, Trade and Industry (METI), the Ministry of Finance (MOF), and the Ministry of Defense, supported by the Cabinet Secretariat's cross-ministerial authority over resource allocation, would convert an administrative forum into a strategic command function.

A priority task for an empowered Headquarters would be to integrate security logic into Japan's relevant health strategies, including the Healthcare Policy led by the Cabinet Secretariat and the Global Health Vision of MHLW, positioning global health as a core pillar of national and regional stability rather than a discretionary line item. Such coherence is particularly critical for managing Japan's engagement with China on health governance, where different ministries currently operate with divergent institutional logics; a unified whole-of-government posture is a prerequisite for constructive engagement with China.

#### Lead regional solidarity against comprehensive health security threats

Building regional solidarity against cross-border health threats requires Japan to convene and sustain the regulatory coalitions through which shared norms are forged. The commercial determinants of health illustrate this logic. The cross-border expansion of tobacco, alcohol, and ultra-processed food industries cannot be curtailed by isolated national action; regional benchmarks are needed to prevent the regulatory arbitrage that undercuts individual countries' efforts. Japan is well placed to convene this harmonisation, but doing so requires confronting the credibility deficit. The disjuncture between Japan's international NCD advocacy and its weak domestic FCTC implementation, including the government's continued stake in Japan Tobacco Inc., must be addressed as a precondition for normative leadership.

Equally pressing is the need for regional governance of AMR. AMR, pandemic preparedness, and pharmaceutical supply-chain security each require cross-border surveillance, regulatory coordination, and shared quality standards.^69^ Existing ASEAN regulatory platforms provide operational foundations for this harmonisation, and Japan is well positioned to anchor their expansion through the Pharmaceuticals and Medical Devices Agency (PMDA), whose regulatory review and post-market safety monitoring experience complements its regional convening capacity. The governance of AI-enabled health technologies presents an analogous cross-border challenge; Japan should build on its leadership of the G7 Hiroshima AI Process to convene regional standards for algorithmic validation, data protection, and accountability,^70^ ensuring that these tools reduce rather than entrench health inequities, while leading coordinated action against health-related misinformation and disinformation to safeguard public trust in evidence-based interventions.

Given China's expanding role in pharmaceutical manufacturing, digital health, and multilateral health governance,^28^ effective regulatory harmonisation in the Western Pacific cannot be achieved without its participation. Japan should pursue issue-based engagement with China within these regulatory frameworks, seeking convergence on technical standards and surveillance protocols where shared interests exist, such as data privacy standards for AI-enabled health tools and vaccine cold-chain protocols, while preserving the rules-based governance principles that underpin regional trust. Achieving these regulatory benchmarks requires reciprocal commitment from regional partners, whose sustained investment in domestic regulatory capacity and data-sharing infrastructure is essential to maintain harmonised standards beyond their initial adoption.

#### Mainstream planetary health through cross-sectoral diplomacy

Planetary resilience requires not only sectoral health interventions but the diplomatic integration of health considerations into the governance of social systems. The national-level evidence on planetary-boundary transgression positions Japan as a country that must both contribute its industrial and disaster-resilience expertise and absorb sustainability lessons from regional partners with a smaller ecological overshoot. A diplomatic task is to translate Japan's hard-won domestic expertise in mitigating industrial pollution and engineering disaster-resilient infrastructure into regional norm-setting.^71^ This entails championing a Health in All Policies (HiAP) approach that operationalises health metrics across sectors, from transport and energy to agriculture and urban planning,^72^ throughout the Western Pacific. HiAP implementation has stalled globally, in large part because health ministries lack the convening power to influence other sectors' agendas. A pragmatic entry point is to build on Japan's existing disaster risk reduction (DRR) partnerships in the region—including the ASEAN Regional Capacity on Health Development (ARCH) Project for ASEAN disaster health management—where health components exist but health metrics have not been systematically integrated across the full range of programming. Embedding such metrics within these established bilateral frameworks offers a lower-cost first step, from which expansion into other sectors can proceed incrementally.

Concretely, Japan should lead the integration of disaster risk reduction into primary healthcare infrastructure, ensuring that health systems can maintain functionality during extreme weather events and compound environmental crises. Recovery standards must move beyond restoring pre-disaster conditions to enforcing build-back-better principles that anticipate future climate-sensitive health risks.

### Recommendation 2: operationalise co-evolution through institutional and digital integration

Recommendation 2 addresses the breakdown of the circular model by building the institutional and digital infrastructure through which circular co-evolution can operate in practice.

#### Foster reciprocal innovation through regulatory co-evolution

Operationalising circular co-evolution requires reinvigorating AHWIN toward the systematic operationalisation of reciprocal innovation. AHWIN's bidirectional ambition has remained largely aspirational because the domestic regulatory caution and stakeholder complexity pose structural barriers to adopting innovations originating abroad, while the Japan International Cooperation Agency (JICA)'s project evaluation framework has historically rewarded outbound technology transfer rather than inbound learning. A regulatory sandbox, drawing on the National Strategic Special Zones framework, could enable controlled piloting of ASEAN-originated health innovations—including mHealth platforms, AI-assisted diagnostics, and community-based care models^73^—within the Japanese system, while the incorporation of reverse learning indicators into JICA programme evaluations would create institutional incentives for genuinely bidirectional exchange. Building on the structural complementarity diagnosed in Fig. 2, transnational innovation ecosystems linking researchers, regulators, and entrepreneurs can co-develop solutions that neither partner could achieve in isolation.

Reciprocally, Japan contributes the regulatory depth essential for long-term resilience. Japan should leverage its comprehensive legislative systems, in NCD control, universal insurance design, and long-term care, to build mutually compatible and safe regulatory environments across the region.^8^ For this reciprocity to function, partner countries would need to systematically document and share their implementation innovations—for example, through case repositories within the UHC Knowledge Hub or joint evaluation programmes—rather than relying on ad hoc exchanges; without such institutional commitment on both sides, the ‘inbound arrow’ of co-evolution will remain aspirational.

#### Lead regional consensus on cross-border health mobility and digital interoperability

Japan should lead the development of a regional digital interoperability framework enabling the secure exchange of health data for shared intelligence.^52^ Given that Japan's own digital health infrastructure remains fragmented and that ASEAN member states vary widely in digital maturity, pragmatic sequencing is essential: the first phase should target cross-border infectious disease surveillance data exchange, building on the existing International Health Regulations (IHR) reporting framework, while full interoperability of digital health records remains a medium-term objective. This digital infrastructure also underpins the portability of health records as workers and patients move across borders, and is essential for advancing the mutual recognition of professional qualifications, building on ASEAN's existing Mutual Recognition Arrangements (MRA) for healthcare professionals and transforming demographic pressures into shared opportunities for human capital circulation. As documented in the companion paper,^20^ realising circular workforce mobility requires moving beyond short-term labour supplementation toward structured pathways for skills development and career reciprocity.

#### Build shared technical infrastructure for climate-resilient regional health response

Whereas the section ‘Mainstream planetary health through cross-sectoral diplomacy’ addresses the transfer of Japan's domestic expertise into regional norm-setting, the institutional task here is distinct: the co-development and deployment of adaptive technologies for climate-resilient health infrastructure as shared physical and operational assets. Japan should leverage its advanced disaster risk reduction expertise and meteorological monitoring capacity to lead the establishment of joint research and development (R&D) platforms for climate-adaptive health technologies, negotiate regional pre-positioning agreements for essential medical supplies in disaster-prone areas, and develop common technical specifications for climate-health early-warning systems that enable interoperable alert and response across national borders.

### Recommendation 3: transition to impact-based financing for accountability and equity

The diagnosis section revealed that the absence of a compelling national-interest narrative leaves global health financing perpetually vulnerable to fiscal retrenchment. Recommendation 3 addresses this accountability deficit by transitioning from volume-based ODA to impact-based financing, reframing global health expenditure as a measurable strategic investment in mutual risk reduction rather than discretionary aid.

#### Evaluate diplomacy via health impact

Evaluation frameworks must shift toward outcome indicators such as disability-adjusted life years (DALYs) averted, UHC service coverage expansion,^12^ and regulatory harmonisation achieved. The methodological challenges are substantial: the fundamental attribution problem, the limitations of Organisation for Economic Co-operation and Development–DAC (OECD-DAC) evaluation timelines,^74^ and the difficulty of isolating Japan's contribution within complex aid ecosystems. Supplementary Panel outlines a three-method framework for addressing these challenges, combining contribution analysis, quasi-experimental methods, and co-produced evaluation, ensuring that evaluation itself becomes an instrument of capacity strengthening rather than an extractive exercise. Furthermore, public R&D funding should incorporate strategic access provisions, encouraging voluntary technology transfer or tiered pricing for low- and middle-income countries,^75^ to ensure that publicly funded innovations contribute to regional health equity while expanding the footprint of Japan's medical sector.

#### Align regional governance with sustainable financing

Japan should leverage its diplomatic standing to align the fiscal scale of multilateral development banks (MDBs), specifically the World Bank and the Asian Development Bank (ADB), with the technical mandates and regional ownership of WHO and diverse regional health frameworks such as ASEAN and the Pacific Islands Forum.^76^^,^^77^ As China's influence in multilateral health financing grows, including through the Asian Infrastructure Investment Bank (AIIB),^78^ Japan's alignment strategy should explicitly address how to engage Chinese-backed institutions within this rules-based framework, for instance through joint financing criteria, shared outcome metrics, and coordinated country diagnosis, ensuring that competing financing channels reinforce rather than fragment regional health governance.

Equally critical is the transition toward domestic resource mobilisation. Japan should support this transition by strengthening public financial management (PFM) and administrative integrity in partner nations, ensuring that domestic health budgets are executed effectively. Critically, the primary agency for this transition lies with partner countries themselves, whose political commitment to progressive domestic health financing, including broadening revenue bases and reducing out-of-pocket expenditure, is the indispensable counterpart to Japan's technical and diplomatic support. The UHC Knowledge Hub should serve as the platform for operationalising these financial governance models—specifically by disseminating approaches that integrate health financing with broader fiscal stewardship, thereby embedding health budgets within national public financial management systems rather than treating them as isolated sectoral expenditures. In its initial phase (2025–2030), the Hub should concentrate on health financing governance, before gradually broadening its mandate to encompass planetary health dimensions.

#### Mobilise resources for climate-health intelligence

Securing international resources for climate-health intelligence systems that provide early warning of environmental anomalies, from heat-related mortality surges to vector-borne disease range expansion, before they escalate into regional crises is essential.^79^ System-building support should target nations with the most limited crisis-response capacity. A regional public-good learning platform, anchored in the UHC Knowledge Hub, should systematically capture and disseminate lessons from both successes and failures in climate-health adaptation, creating the institutional memory that the region currently lacks.

## Conclusion

The structural barriers diagnosed here, a crisis of solidarity in the multilateral architecture, the domestic political economy of episodic leadership, and the limits of unidirectional knowledge transfer, are not unique to Japan; ageing economies across the Western Pacific face analogous constraints, including fiscal pressure on social systems, rising NCD burdens, and the imperative to reconcile domestic priorities with global responsibilities. Japan's strategic pivot toward human security diplomacy offers more than a national reform agenda. It demonstrates how health can be elevated from discretionary expenditure to the level of high politics through norm-setting and institutional leadership rather than geopolitical leverage, ensuring commitment that outlasts individual summits and electoral cycles. The circular model of co-evolution, in which domestic resilience and regional solidarity reinforce one another, redefines national interest within planetary boundaries. This synthesis is offered to inform the policy dialogue and collective engagement across the Western Pacific, on which the institutional will to sustain this transformation depends.

## Contributors

SN conceptualised the review and developed the analytical framework. HS, LY, and SN developed the methodology and conducted the thematic analysis of the authors’ responses to the expert consultation. HS, LY, and SN accessed and verified the underlying materials and data used in the analysis, including consultation responses, coded thematic outputs, and literature-derived evidence. HS and SN drafted the original manuscript. All authors contributed to the interpretation of findings and provided critical input on the analytical content. HS and SN prepared the figures and tables. YN, TS, and SN coordinated project administration. SN supervised the review and acquired funding. All authors critically revised the manuscript for important intellectual content, approved the final version, and agree to be accountable for all aspects of the work. SN is the corresponding author.

## Declaration of generative AI and AI-assisted technologies in the writing process

During the preparation of this manuscript, the authors used Claude (Anthropic, San Francisco, CA, USA) in order to improve the readability and language of the manuscript. After using this tool, the authors reviewed and edited the content as needed and take full responsibility for the content of the publication.

## Declaration of interests

Keizo Takemi is chair of the Asian Population and Development Association and served as Japan's Minister of Health, Labour and Welfare from 2023 to 2024. Ataru Igarashi reports grants or contracts from Moderna and Takeda Pharmaceutical Company Limited, consulting fees from PhRMA Japan, EFPIA Japan, and the Japan Pharmaceutical Manufacturers Association, and honoraria from the Ministry of Health, Labour and Welfare of Japan, outside the submitted work. All other authors declare no competing interests. The views expressed in this Review are those of the authors and do not necessarily represent those of their institutions.

## Appendix A Supplementary data

Supplementary Material

Translated Abstract
