# Supplementary material for: Japan's global health engagement in the Western Pacific: diagnosing structural inertia and a framework for human security diplomacy
Source: Lancet Reg Health West Pac. 2026 Jul 15;72:101921. doi: 10.1016/j.lanwpc.2026.101921 (PMC13478820; doi:10.1016/j.lanwpc.2026.101921)
Supplement: Supplementary file 1 [file mmc1.pdf]

Supplementary Appendix 1

Japan's global health engagement in the Western Pacific: diagnosing structural inertia and a framework for human security diplomacy

Contents

Supplementary Methods: Expert working group consultation: procedures and survey instruments

Supplementary Table 1: Round 2: integrated audit trail from coded concepts to Vision 2040 goals, the goal-action matrix (Table 2), and representative statements

Supplementary Panel: Evaluating Japan's contribution in complex aid ecosystems

Supplementary Methods: Expert working group consultation: procedures and survey instruments

Section 1. Working group composition

The working group had 25 members, including academics, diplomats, and development practitioners from Japan and the Western Pacific region, with attention to a mix of senior and early-career members and gender balance. The following table shows the composition by institutional type and regional background.

Working group composition

Dimension	Category	n
Institutional type	Academic institutions	15
	Multilateral / intergovernmental organisations and regional think tanks	4
	Policy implementation bodies and government-affiliated institutions	6
Regional background	Japan	19
	Other countries (China, Singapore, Thailand, Myanmar, Nepal, Germany)	6

Section 2. Consultation procedure

2.1 Two-round design rationale

The consultation ran in two rounds with different purposes. Round 1, the Challenge Mapping round, asked authors to identify structural challenges confronting Japan's health system and its global health strategy. Round 2, the Goal and Action Mapping round, took place after the Round 1 results had been synthesised and shared with the working group. In Round 2, authors set out forward-looking goals and concrete recommendations linked to the Round 1 challenges. Running the rounds in sequence ensured that the Round 2 recommendations rested on the diagnosis produced in Round 1.

2.2 Round 1 procedure and response format

Round 1 took place in September 2025. Authors were asked to give up to three challenges in each of three dimensions: the international dimension covered global health financing, governance, and institutional design. The domestic dimension covered issues rooted in Japan's own systems and operations. The interface dimension covered the policy space between international initiatives and domestic systems. Entries were capped at 150 words,

and authors were asked to add a brief note on background or rationale. Section 3 reproduces the full Round 1 instrument.

2.3 Round 2 procedure and response format

Round 2 took place in November 2025 after the synthesised Round 1 results had been shared with the working group. Authors were asked to give up to three goals and up to three actions, each capped at 150 words. For every entry, respondents also ticked one or more of six boxes indicating which of the Round 1 challenges the entry addressed. The six boxes were labelled A-1 to A-3 for global health and B-1 to B-3 for domestic health. Section 4 reproduces the full Round 2 instrument.

2.4 Coding and consensus procedure

Responses were analysed using inductive thematic analysis, through six iterative stages: familiarisation, initial coding generated directly from the data, theme generation, theme review, theme definition and naming, and assessment of thematic convergence. One author conducted the initial coding of responses, and two authors subsequently reviewed and verified the coding. The consolidated themes and their interpretation were then discussed across all authors to reach consensus. Codes were then grouped into higher-order themes. In Round 1, themes fell into the three dimensions used in the instrument, namely international, domestic, and interface, and were grouped further into six structural challenge categories: three for global health (barriers A, B, C in Table 1) and three for domestic health. The three global-health categories correspond to barriers A, B, and C diagnosed in Table 1 of the main text, while the domestic-health categories informed the companion paper on Japan's domestic health-system reform. In Round 2, each response was first classified as either a goal, meaning a desired end-state, or an action, meaning a step for reaching that state, and coded on that basis. The resulting codes were consolidated into the goal-action matrix in Table 2 of the main text, which links each diagnosed challenge to the strategic recommendations proposed for it.

2.5 Working group meetings

The working group convened twice in plenary during the consultation. The first meeting, held in October 2025 between the two rounds, reviewed the synthesised Round 1 findings and aligned on the framing for Round 2. The second meeting, held in December 2025 after Round 2, reviewed the synthesised goals, actions, and the resulting goal–action matrix, and provided an opportunity to refine the diagnosed challenges and the wording of the recommendations presented in the main text. The analytical audit trail linking Round 2 coded concepts to the three strategic goals and the cells of the goal–action matrix is provided in Supplementary Table 1. Representative anonymised statements from Round 2,

grouped by goal, are also provided in Supplementary Table 1.

Section 3. Round 1 survey instrument: Challenge Mapping Survey

The following reproduces the Round 1 survey instrument as administered to authors in September 2025.

Guidelines for Response

Please categorise your challenges under the three themes below. Within your professional scope, please provide up to three challenges, with each entry not exceeding 150 words. Where possible, please include a brief explanation of the background or rationale.

- **International Dimension:** Challenges related to global health financing, governance, and institutional design, where Japan faces difficulties or where Japan's role is at stake amid international developments.
- **Domestic Dimension:** Challenges rooted in Japan's own systems and operations, such as demographic change, healthcare financing, workforce shortages, and system reforms.
- **Interface between International and Domestic Dimensions:** Challenges arising at the policy interface, such as insufficient translation of international initiatives into domestic systems or limited utilisation of domestic knowledge and reforms to build international trust, both of which hinder a two-way cycle.

Examples

Clear challenges

- Declining global health financing raises concerns about the sustainability of health system strengthening in low- and middle-income countries.
- Institutional and financing structures in Japan are under increasing strain, raising concerns about the long-term sustainability of universal health coverage.
- While the international circulation of healthcare professionals is advancing, it has not sufficiently alleviated domestic workforce shortages.

Unclear challenges

- "How can more funding be mobilised?" (framed as a question rather than a challenge)
- "The UHC Knowledge Hub should be strengthened" (expressed as a solution rather than a challenge)
- "Perhaps AI could be used" (a proposal without a clearly defined challenge)

The challenges collected will provide the starting point for subsequent deliberations on "vision" and "action."

Response Page (Challenge Mapping Survey)

Within your professional scope, please list up to three challenges for each category below. Each entry should not exceed 150 words. Where possible, please provide a concise explanation of the background or rationale.

1. International Dimension

Challenges related to global health financing, governance, and institutional design, where Japan faces difficulties or where Japan's role is at stake amid international developments.

Challenge 1:

[response field]

Challenge 2:

[response field]

Challenge 3:

[response field]

2. Domestic Dimension

Challenges inherent to Japan's own systems and operations, such as population ageing, healthcare financing, workforce shortages, and system reforms.

Challenge 1:

[response field]

Challenge 2:

[response field]

Challenge 3:

[response field]

3. Interface between International and Domestic Dimensions

Challenges arising at the policy interface, such as insufficient translation of international initiatives into domestic systems or limited utilisation of domestic knowledge and reforms to build international trust, both of which hinder a two-way cycle.

Challenge 1:

[response field]

Challenge 2:

[response field]

Challenge 3:

[response field]

Optional Free-Entry Section

Please add any additional comments or related points you consider relevant.

Section 4. Round 2 survey instrument: Goal and Action Mapping Survey

The following reproduces the Round 2 survey instrument as administered to authors in November 2025.

Guidelines for Response: Defining "Goals" and "Actions"

To ensure clarity and consistency, please adhere to the following definitions, which distinguish between the desired future state (Goal) and the strategic steps to get there (Action).

Part 1: GOALS (The "What")

Definition: A "Goal" (which we previously termed "Vision") defines "What is the desired future state we should aim for?".

Explanation: This is not a specific, granular solution. Instead, it should be a compelling, high-level depiction of the new paradigm or the ideal state we want to achieve once the Structural Challenges have been overcome. It describes the destination.

Examples of a GOAL:

Clear Examples (what we want):

"A resilient and adaptive 'learning' health system that institutionalises the integration of global best practices."

"A proactive and consistent Japanese global health posture that shapes new international norms on health and security."

Unclear Examples (what to avoid):

"Japan should establish an independent HTA body." (This is an Action.)

"Japan's leadership is too episodic." (This is a Challenge.)

Part 2: ACTIONS (The "How")

Definition: An "Action" defines "How do we get there?".

Explanation: This is a concrete, high-level strategic recommendation required to move from the current Challenge to the desired Goal. It is the vehicle, not the destination.

Examples of an ACTION:

Clear Examples (what we want):

- "Establish a permanent, cross-ministerial body linking health to economic security to enable proactive norm-setting."
- "Shift resource allocation from an acute, hospital-centric model to integrated, community-based care for multi-morbidity."

Unclear Examples (what to avoid):

"Japan's system should be more sustainable." (This is a Goal.)

"The fee-for-service model is inefficient." (This is a Challenge.)

Response Page (Goal and Action Mapping Survey)

Within your professional scope, please list up to three (3) Goals and up to three (3) Actions. Each entry should not exceed 150 words. For each entry, please check the box(es) corresponding to the primary Structural Challenge(s) it addresses.

Part 1: GOALS (The "What")

What is the desired future state we should aim for?

Goal 1:

[response field; max 150 words]

Related Structural Challenge(s) (please check all that apply):

☐ A-1: The Shifting Global Health Architecture and the Crisis of Solidarity

☐ A-2: Japan's Fragmented Policy and the "Episodic" Nature of its Leadership

☐ A-3: Breakdown in the Circular Model: Missed Opportunities for Mutual Growth

☐ B-1: A Systemic Mismatch with a New Era of Health Challenges

☐ B-2: An Outdated Infrastructure Inhibiting a Paradigm Shift

☐ B-3: Insularity and Failure to Leverage Global Interdependence

Goal 2:

[response field; max 150 words]

Related Structural Challenge(s) (please check all that apply):

☐ A-1: The Shifting Global Health Architecture and the Crisis of Solidarity

☐ A-2: Japan's Fragmented Policy and the "Episodic" Nature of its Leadership

☐ A-3: Breakdown in the Circular Model: Missed Opportunities for Mutual Growth

☐ B-1: A Systemic Mismatch with a New Era of Health Challenges

☐ B-2: An Outdated Infrastructure Inhibiting a Paradigm Shift

☐ B-3: Insularity and Failure to Leverage Global Interdependence

Goal 3:

[response field; max 150 words]

Related Structural Challenge(s) (please check all that apply):

☐ A-1: The Shifting Global Health Architecture and the Crisis of Solidarity

☐ A-2: Japan's Fragmented Policy and the "Episodic" Nature of its Leadership

☐ A-3: Breakdown in the Circular Model: Missed Opportunities for Mutual Growth

☐ B-1: A Systemic Mismatch with a New Era of Health Challenges
☐ B-2: An Outdated Infrastructure Inhibiting a Paradigm Shift
☐ B-3: Insularity and Failure to Leverage Global Interdependence

Part 2: ACTIONS (The "How")

What concrete, high-level strategic recommendations are necessary to achieve these Goals?

Action 1:

[response field; max 150 words]

Related Structural Challenge(s) (please check all that apply):

☐ A-1: The Shifting Global Health Architecture and the Crisis of Solidarity
☐ A-2: Japan's Fragmented Policy and the "Episodic" Nature of its Leadership
☐ A-3: Breakdown in the Circular Model: Missed Opportunities for Mutual Growth
☐ B-1: A Systemic Mismatch with a New Era of Health Challenges
☐ B-2: An Outdated Infrastructure Inhibiting a Paradigm Shift
☐ B-3: Insularity and Failure to Leverage Global Interdependence

Action 2:

[response field; max 150 words]

Related Structural Challenge(s) (please check all that apply):

☐ A-1: The Shifting Global Health Architecture and the Crisis of Solidarity
☐ A-2: Japan's Fragmented Policy and the "Episodic" Nature of its Leadership
☐ A-3: Breakdown in the Circular Model: Missed Opportunities for Mutual Growth
☐ B-1: A Systemic Mismatch with a New Era of Health Challenges
☐ B-2: An Outdated Infrastructure Inhibiting a Paradigm Shift
☐ B-3: Insularity and Failure to Leverage Global Interdependence

Action 3:

[response field; max 150 words]

Related Structural Challenge(s) (please check all that apply):

☐ A-1: The Shifting Global Health Architecture and the Crisis of Solidarity
☐ A-2: Japan's Fragmented Policy and the "Episodic" Nature of its Leadership
☐ A-3: Breakdown in the Circular Model: Missed Opportunities for Mutual Growth
☐ B-1: A Systemic Mismatch with a New Era of Health Challenges
☐ B-2: An Outdated Infrastructure Inhibiting a Paradigm Shift
☐ B-3: Insularity and Failure to Leverage Global Interdependence

Optional Free-Entry Section

Please add any additional comments or related points you consider relevant.

Supplementary Table 1: Round 2: integrated audit trail from coded concepts to Vision 2040 goals, the goal-action matrix (Table 2), and representative statements

This table integrates the Round 2 audit trail across three dimensions for each of the three Vision 2040 goals: (i) the principal structural barrier from Table 1 that the goal addresses, together with the principal Round 2 thematic clusters that fed into the goal; (ii) the action items distributed across the three action domains (Human security diplomacy; Institutional and digital integration; Impact-based financing), corresponding to the three columns of Table 2 of the main text; and (iii) representative anonymised statements illustrating the working group's collective position on the goal. Reading down each goal block recovers the full derivation from coded concepts to the corresponding row of Table 2.

Audit-trail dimension	Content
**Goal 1: Harmonise global contribution with domestic growth through human security diplomacy**	
**Structural barrier (Table 1) addressed**	Barrier B: Japan's fragmented policy and the structural roots of episodic leadership.
**Principal Round 2 thematic clusters**	• Reframing of global health as integral to economic security<br>• Whole-of-government coordination and upgrading of the Headquarters for Healthcare Policy<br>• Protected multi-year budget envelopes insulated from fiscal retrenchment<br>• Reciprocal innovation and reverse learning via revitalised AHWIN<br>• Pipeline and career-path reform for internationally engaged professionals
**Action domain 1 — Human security diplomacy (Table 2, column 1)**	• Upgrade Headquarters for Healthcare Policy with budget coordination authority<br>• Cross-ministerial personnel exchange (MOFA, MHLW, METI, MoF)<br>• Fuse economic security logic into health strategies
**Action domain 2 — Institutional and digital integration (Table 2, column 2)**	• Revitalise AHWIN for reciprocal innovation<br>• Regulatory sandbox for ASEAN-originated innovations<br>• Reverse-learning indicators in JICA evaluation

Audit-trail dimension	Content
	• Transnational innovation ecosystems
**Action domain 3 — Impact-based financing (Table 2, column 3)**	• Shift evaluation from inputs to outcomes (DALYs averted, UHC coverage, regulatory harmonisation)<br>• Long-term trajectory focus to address attribution<br>• Strategic access provisions in public R&D funding
**Representative anonymised statements (Round 2)**	• *Global health must be reframed as integral to economic security, not as discretionary aid.*<br>• *Regional health security is a public good whose under-provision directly threatens domestic economic stability through supply-chain exposure.*<br>• *The Headquarters for Healthcare Policy needs binding budget authority, not only a coordination mandate.*<br>• *Reverse-learning indicators must be embedded in evaluation frameworks if inbound flows are to materialise.*
**Goal 2: Foster solidarity and regional public goods through catalytic leadership**	
**Structural barrier (Table 1) addressed**	Barrier A: The shifting global health architecture and the crisis of solidarity.
**Principal Round 2 thematic clusters**	• Regional regulatory harmonisation for commercial determinants of health<br>• Cross-border governance of antimicrobial resistance and pandemic preparedness<br>• Pharmaceutical supply-chain quality standards and shared manufacturing protocols<br>• AI governance building on the G7 Hiroshima AI Process<br>• Coordinated action against health-related misinformation<br>• Digital interoperability and workforce mobility via ASEAN MRAs<br>• Issue-based engagement with China within rules-based governance

Audit-trail dimension	Content
**Action domain 1 — Human security diplomacy (Table 2, column 1)**	• Regional regulatory harmonisation for commercial determinants of health<br>• ASEAN PPWG mandate extension to AMR stewardship<br>• PMAS evolution to proactive pharmacovigilance<br>• G7 Hiroshima AI Process-based regional AI standards<br>• Misinformation countermeasures
**Action domain 2 — Institutional and digital integration (Table 2, column 2)**	• Regional digital interoperability framework<br>• Portability of health records across borders<br>• Advancement of ASEAN MRAs for professional mobility<br>• Circular workforce mobility as regional public good
**Action domain 3 — Impact-based financing (Table 2, column 3)**	• Align MDB fiscal scale (World Bank, ADB) with WHO technical mandates<br>• Engage AIIB within rules-based framework<br>• Strengthen PFM and administrative integrity in partner nations<br>• UHC Knowledge Hub for financial governance models
**Representative anonymised statements (Round 2)**	*• Japan should convene the regional regulatory coalitions that prevent regulatory arbitrage across borders.*<br>*• AMR and pandemic preparedness require cross-border architecture that no single country can build alone.*<br>*• Engagement with China must be issue-based and rules-preserving, neither confrontational nor accommodating.*<br>*• The G7 Hiroshima AI Process provides a foundation on which regional health-AI governance can be built.*
**Goal 3: Mainstream health into social systems for planetary resilience**	

Audit-trail dimension	Content
**Structural barrier (Table 1) addressed**	Barrier C: Breakdown in the circular model; cross-sectoral dimensions extending beyond the health sector.
**Principal Round 2 thematic clusters**	• Integration of disaster risk reduction into primary healthcare infrastructure<br>• Health in All Policies across transport, energy, agriculture, and urban planning<br>• Climate-health early-warning systems as regional public goods<br>• Joint R&D platforms for climate-adaptive health technologies<br>• Regional pre-positioning of essential medical supplies<br>• UHC Knowledge Hub as a learning platform for climate-health adaptation
**Action domain 1 — Human security diplomacy (Table 2, column 1)**	• Integration of DRR into primary healthcare infrastructure<br>• Health in All Policies across transport, energy, agriculture, urban planning<br>• Embed health metrics in ARCH Project
**Action domain 2 — Institutional and digital integration (Table 2, column 2)**	• Joint R&D platforms for climate-adaptive health technologies<br>• Regional pre-positioning of essential medical supplies<br>• Common technical specifications for climate-health early warning
**Action domain 3 — Impact-based financing (Table 2, column 3)**	• International resource mobilisation for climate-health intelligence<br>• Targeted system-building for low-capacity nations<br>• UHC Knowledge Hub as learning platform for climate-health adaptation
**Representative anonymised statements (Round 2)**	*• Health ministries cannot drive Health in All Policies alone; the lead must come from the Cabinet level.*<br>*• Disaster risk reduction is a natural entry point because Japan already has partnerships and credibility in the region.*

Audit-trail dimension	Content
	• Climate-health intelligence systems are a regional public good that Japan is uniquely positioned to anchor.<br>*• Build-back-better principles must be extended beyond infrastructure to health-system recovery standards.*

Note. The mapping from Round 2 coded concepts to Vision 2040 goals and to action items in Table 2 was finalised through iterative working-group discussion. Statements in the 'Representative anonymised statements' row are paraphrased to prevent attribution and are released with prior participant consent.

Supplementary Panel: Evaluating Japan's contribution in complex aid ecosystems

When multiple donors, domestic reforms, and secular trends simultaneously shape health outcomes, no single actor can claim sole attribution. The question for Japan's official development assistance (ODA) evaluation is not whether its programmes produced measurable outputs—existing evaluations already do this—but whether sustained engagement in institutional design, workforce development, and health financing governance made a credible contribution to long-term population-level outcomes. Three complementary methods address this question.

- Contribution analysis provides the backbone. Rather than isolating Japan's effect through experimental control, it assembles structured evidence to assess whether an intervention plausibly contributed to observed outcomes, systematically testing a causal narrative against data and ruling out rival explanations.[1] When combined with outcome harvesting, which works backwards from observed changes to identify what contributed to them, the approach can also capture unintended pathways of influence—a critical capability given that Japan's system-level investments often generate effects through indirect and non-linear routes.[2] Now widely adopted in multilateral and bilateral development evaluation—including by the Global Fund,[2] the United Nations Development Programme (UNDP),[3] the Foreign, Commonwealth and Development Office (FCDO),[4] and the Deutsche Gesellschaft für Internationale Zusammenarbeit (GIZ),[5] —contribution analysis is compatible with the Japan International Cooperation Agency (JICA)'s existing qualitative ex-post assessments.
- Quasi-experimental methods provide quantitative rigour where data permit. Difference-in-differences and synthetic control designs, applied to routine health information system data, can estimate effect sizes for strategically selected programmes—particularly long-running health system strengthening initiatives where subnational variation and time-series data exist. The World Bank's Development Impact Evaluation group has institutionalised these methods in development evaluation;[6] Gavi's post-transition assessments used synthetic control designs to measure sustained programme impact after donor withdrawal.[7] These methods are not feasible for every project but generate high-value evidence for Japan's most significant investments.
- Co-produced evaluation with partner-country institutions ensures both quality and sustainability. Co-designing evaluation with national health authorities and integrating data collection into platforms such as the District Health Information Software 2 (DHIS2) builds the local analytical capacity that credible evaluation depends on—converting the evaluation process itself into system strengthening, consistent with Japan's emphasis on ownership.

References:

1. Mayne J. Contribution analysis: Coming of age? *Evaluation* 2012; **18**: 270–80.
2. The Global Fund to Fight AIDS T and M. Evaluation of Community Responses and Systems Strengthening. 2024. https://www.theglobalfund.org/media/2vyohrxi/iep_gf-elo-2024-07_report_en.pdf (accessed May 7, 2026)
3. Independent Evaluation Office U. CONTRIBUTION ANALYSIS. https://erc.undp.org/methods-center/methods/evaluation-methods/contribution-analysis (accessed May 7, 2026)
4. Foreign C& DO. FCDO annual evaluation report 2023 to 2024. 2025. https://www.gov.uk/government/publications/fcdo-annual-evaluation-report-2023-to-2024/fcdo-annual-evaluation-report-2023-to-2024 (accessed May 7, 2026)
5. Deutsche Gesellschaft für International Zusammenarbeit (GIZ). GIZ's evaluation system. 2022. https://www.giz.de/en/downloads/giz-2023-en-gizs-evaluation-sytem-central-project-evaluations-in-bmz-business.pdf (accessed May 7, 2026)
6. World Bank. Science for Impact - better evidence for better decisions. 2019. https://documents1.worldbank.org/curated/en/942491550779087507/pdf/Science-for-Impact-Better-Evidence-for-Better-Decisions-The-Dime-Experience.pdf (accessed May 7, 2026)
7. Kolesar RJ, Spruk R, Tsheten T. Evaluating Country Performance After Transitioning From Gavi Assistance: An Applied Synthetic Control Analysis. *Glob Health Sci Pract* 2023; **11**: e2200536.
