# Supplementary material for: Japan's global health engagement in the Western Pacific: diagnosing structural inertia and a framework for human security diplomacy
Source: Lancet Reg Health West Pac. 2026 Jul 15;72:101921. doi: 10.1016/j.lanwpc.2026.101921 (PMC13478820; doi:10.1016/j.lanwpc.2026.101921)
Supplement: Supplementary file 2 [file mmc2.docx]

**Supplementary Appendix**

Translated abstract in Japanese

Japan’s global health engagement in the Western Pacific: diagnosing structural inertia and a framework for human security diplomacy

*Editor’s note: This translation in Japanese was submitted by the authors and we reproduce it as supplied. It has not been peer reviewed. Our editorial processes have only been applied to the original abstract in English, which should serve as reference for this manuscript.*

国際保健ガバナンスは、国際秩序の多極化が進む中で分断を深めている。保健分野への開発援助は縮小し、地政学的な分断によって国際社会の協調行動は停滞し、保健は各国の裁量に左右される対外援助の一分野にとどまっている。アジアで唯一のG7構成国であり、ユニバーサル・ヘルス・カバレッジの先駆的な担い手でもある日本は、国内の社会保障制度を維持する必要性と、急速に変化する国際環境への対応との間で緊張に直面している。本稿では、二度にわたる専門家協議と文献統合に基づき、戦略的停滞の循環を形成する三つの相互に関連した構造的障壁を診断する。第一に、多国間体制において連帯が弱まり、資金が縮小し、ガバナンスが機能不全に陥りつつあることである。第二に、日本国内の制度的分断とインセンティブの不整合によって、国際保健におけるリーダーシップが高い政治的関心を集める局面に偏り、持続的な関与につながりにくいことである。第三に、日本から地域への知識移転に偏り、地域から日本への学びが十分に制度化されていないため、国内の能力と地域連携を再活性化する機会が制約されていることである。我々は、保健を裁量的支出ではなく地域アーキテクチャーの柱として位置づける「人間の安全保障外交」の枠組みを提案する。この枠組みは、三つの目標を掲げる。すなわち、国際貢献と国内成長の調和、触媒的リーダーシップを通じた連帯と地域公共財の形成、そしてプラネタリー・レジリエンスに向けて保健を社会システムに組み込むことである。その中核にあるのは「循環的共進化」であり、日本の制度的知見とパートナー国の革新が相互に補強し合う関係を指す。
